# Punicalagin with anti-inflammatory activities affects Brd-4 mediated chromatin remodeling for attenuating inflammatory osteolysis

**DOI:** 10.1038/s41598-026-41262-3

**Published:** 2026-03-10

**Authors:** Huiping Li, Qilin Li, Tianhao Wan, Yexin Wang, Shanyong Zhang

**Affiliations:** Department of Oral Surgery, Shanghai Ninth People’s Hospital, Shanghai Jiao Tong University School of Medicine; College of Stomatology, Shanghai Jiao Tong University; National Center for Stomatology; National Clinical Research Center for Oral Diseases; Shanghai Key Laboratory of Stomatology, Shanghai, 200011 China

**Keywords:** Punicalagin, Osteolysis, Osteoclast, Brd4, Inflammatory macrophage, Biochemistry, Cell biology, Diseases, Drug discovery, Immunology, Molecular biology

## Abstract

Inflammatory osteolysis is primarily characterized by an extensive macrophage-mediated inflammatory response coupled with osteoclast (OC) formation, triggered by bacterial byproducts and/or environmental stressors. And Osteoarthritis (OA) is one of the most common degenerative diseases in clinical medicine. Currently, anti-inflammatory drugs and intra-articular drug injection are mainly used, but the treatments only relieve symptoms. Punicalagin (PUN), a hydrolyzable tannin derived from pomegranate extract, the suppression of pro-inflammatory cytokine production in macrophages. The therapeutic potential of PUN in alleviating inflammatory osteolysis remains inadequately elucidated. PUN demonstrated favourable biocompatibility and therapeutic potential in vitro, including potent anti-osteoclastic activity, ROS scavenging capacity, and epigenetic regulatory functions. PUN was found to inhibit bromodomain-containing protein 4 (Brd4)-mediated chromatin space remodeling, consequently upregulating the production of endogenous anti-inflammatory factors and antioxidant factors. This study reveals a new therapeutic mechanism that PUN exerts anti-inflammatory effects and regulates epigenetic regulation by influencing Brd4-mediated chromatin remodeling. These findings showed the therapeutic potential of PUN for inflammatory diseases, especially inflammatory osteolysis. Notably, our work identifies a new strategy that synergistically combines osteoclast inhibition with epigenetic regulation, providing a promising direction for the therapies for bone-related inflammatory diseases.

**Supplementary Information:**

The online version contains supplementary material available at 10.1038/s41598-026-41262-3.

## Introduction

In bone-related inflammatory diseases, including osteomyelitis, rheumatoid arthritis (RA), periodontitis, peri-implant inflammation and septic arthritis, osteolysis usually occurs around the area of inflammation and is characterized by infiltration of numerous OCs and immune cells^1,2^. Osteoarthritis (OA), one of the most common degenerative diseases, is characterized by progressive cartilage degeneration, subchondral bone remodeling, osteophyte formation, synovial inflammation, and structural alterations of the joint capsule, ligaments, and associated muscles^3–6^. Disruption of balance between osteoblast (OBs) and OCs accounts for the pathogenesis of OA, and OCs play an important role during this period. Meanwhile, there are many factors contributing to osteolysis. Several studies have shown that inflammatory cytokines, such as interleukin-1β (IL-1β), interleukin-6 (IL-6), tumor necrosis factor-α (TNF-α), mainly expressed by active macrophages chronically, have an essential role for osteolysis. These inflammatory factors further promote the differentiation of macrophages into OCs ^7–10^. It has been reported that NF-κ B ligand receptor activator (RANKL) and TNF-α play a synergistic role in osteoclastogenesis, with low RANKL levels directly stimulating OC precursors to become mature osteoclasts^11,12^. Given this interdependent and mutually reinforcing relationship between inflammatory cells and RANKL, blocking the inflammatory response and OC generation in the treatment of inflammatory bone resorption is critical^13^. Therefore, current strategies for the treatment of inflammatory bone resorption are to inhibit the differentiation of inflammatory macrophages into OCs, as well as reduce the release of inflammatory factors. Conservative treatment of OA, such as non-steroidal anti-inflammatory drugs (NSAIDs), can temporarily relieve pain, but systemic administration often leads to adverse gastrointestinal and cardiovascular reactions, so its clinical application is somewhat limited^14–16^. Intra-articular drug injection is also limited due to the unique cavity structure of the joint, which presents significant challenges to effective drug delivery, as well as the complex production process, immune rejection, and uncertain treatment outcomes^17^.Therefore, it is necessary to further understand osteolysis and find innovative agents.

During OCs differentiation, the metabolic process of mitochondrial oxidative phosphorylation (OXPHOS) is transformed, resulting in the local production of excessive reactive oxygen species (ROS), including peroxides and superoxide radicals^18^. These ROS, as by-products of mitochondrial respiration, can lead to genetic and epigenetic changes^19^. Epigenetics refers to the mechanism by which the genome changes gene expression without changing the original DNA sequence. The three main mechanisms regulated by epigenetics include chemical modification of DNA, post-translational modification of histones, and regulation of non-coding regulatory RNA^20^. Unlike genetic mutations, epigenetic changes are potentially reversible. Evidence shows that dysregulation of numerous essential cartilage molecules is caused by aberrant epigenetic regulatory mechanisms, and it contributes to the development and progression of OA^21^. Studies have shown that joint homeostasis is highly dependent on epigenetic control mechanisms, mainly through post-translational histone modifications, which maintain the molecular properties of articular chondrocytes^22,23^. Among them, acetylation of lysine residues on histones usually opens chromatin for gene transcription and plays a key role in inflammation^24,25^.

In OA, histone acetylation is associated with overactivation of inflammatory pathways ^26^. The bromine domain (Brd), a conserved structural block found in chromatin and transcription-associated proteins, is considered the first chromatin “reader”, present in many chromatin and transcription-associated proteins^27^, and has now been identified as the fundamental mechanism by which histone acetylation regulates cellular gene transcription in response to physiological and environmental signals in transaction. BET proteins including Brd2, Brd3, and Brd4 regulates the expression of inflammatory genes by recognizing the acetylation sites of the genes^28,29^. Brd4 can increase the transcriptional activity of RNA polymerase II by reading histone acetylation and binding to other proteins such as P-TEFb protein for phosphorylation, thus activating the expression of downstream genes ^30^. Effective Brd inhibitors JQ1 and I-BET762 have been reported to selectively target Brd of bromine domain and outer end (BET) family proteins, effectively blocking cell proliferation of testicular nuclear protein (NUT) midline carcinoma, and inhibiting the expression of inflammatory genes in lipopolysaccharide-induced endotoxin shock and bacteria-induced sepsis activated macrophages^31–34^. Therefore, inhibiting the effect of Brd4 can suppress chromatin spatial remodeling and thereby suppress the expression of inflammatory genes.

In recent years, the pharmacological activity of natural compounds has become the focus of human disease research^35^. Compared to synthetic drugs, natural compounds derived from plants have lower cost, higher bioavailability, and less toxicity^36^. Punicalagin (2,3- (S) -Hexahydroxydiphenyl -4,6-(S) -Gallagyl-D-glucose, PUN) is a polyphenol extracted from pomegranate fruit with anti-inflammatory, antitumor and antioxidant properties. And can inhibit oxidative damage of DNA, at the same time, it also has anti-mutation and anti-proliferation effects, is an important anticancer drug^37,38^. Olajide et al. found that PUN could inhibit the neuroinflammation of LPS-activated microglia^39^. In addition, Punicalagin down-regulates M1 macrophages and pyrodeath via the NF-κB signaling pathway, which not only improves arthritis symptoms and subchondral fractures, but also reduces systemic bone loss without toxic effects on the liver or kidney. Xu et al. found that PUN treatment could significantly reduce the production of NO, prostaglandin E2 (PGE2), interleukin (IL)-1β, IL-6 and tumor necrosis factor (TNF)-α induced by LPS-induced RAW264.7 cells^40^. However, no studies have shown the effect of PUN on apparent regulation. In this study, PUN demonstrated excellent epigenetic regulatory function by inhibiting the expression of Brd4, affecting chromatin spatial structure connectivity and activating endogenous anti-inflammatory and antioxidant system of inflammatory macrophages. Our in vivo experiments further validated the efficacy of PUN in alleviating bone resorptive associated with LPS-induced inflammatory osteolysis.

## Results

### Biocompatibility of PUN

**Fig. 1:**
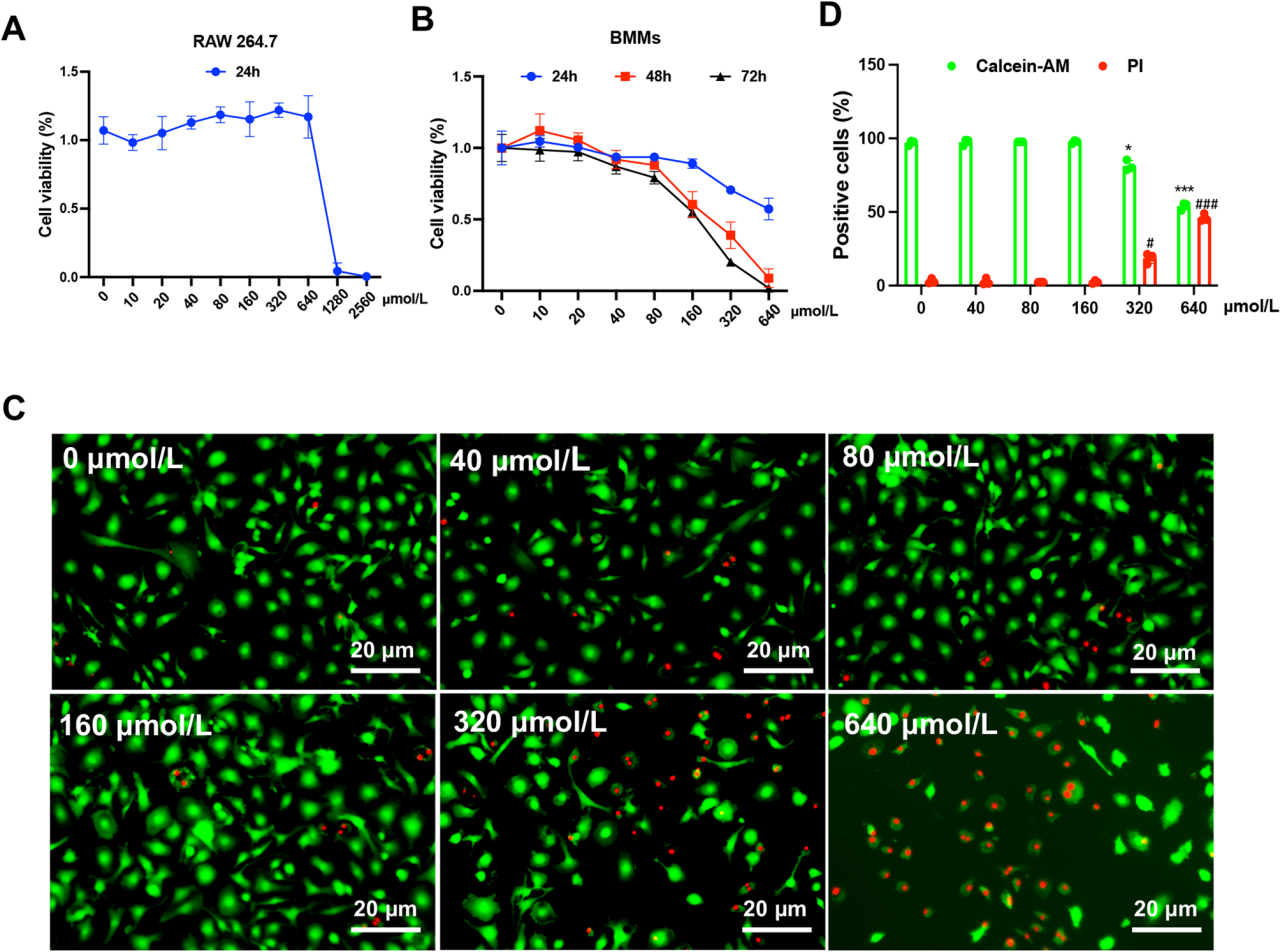
Biocompatibility of PUN. (**A**) Cell viability of RAW264.7 treated with various concentrations of Punicalagin for 24 h. (**B**) Cell viability of BMMs treated with various concentrations of Punicalagin for 24, 48 and 72 h. (**C**) Live/dead staining of BMMs treated with various concentrations of Punicalagin 24 h (**D**) Quantitative analysis of Calcein-AM-positive cells and PI-positive cells. (mean ± SD, one-way ANOVA with Tukey’s multiple comparison test, n = 3 independent samples, *p < 0.05; **p < 0.01; ***p < 0.005; ****p < 0.001).

To assess the biocompatibility and anti-osteoclast effects of PUN, we performed CCK-8 and CalceinAM/Propidiumiodide (Calcein-AM/PI) staining in BMMs and RAW 264.7 macrophages. Cells exhibit high viability following treatment of various concentration of PUN, even at concentration of 320 μmol/L for 24 h (Fig. 1A). For BMMs, PUN was well tolerated when concentration was up to160μmol/L for 24 h. However, significant cytotoxicity was observed after 48 h or 72 h at this concentration.

For longer duration, concentration up to 80 μmol/L remain safe (Fig. 1B). With increasing concentration of PUN, the proportion of PI-positive RAW 264.7 macrophages increased, coupled with reduction of Calcein-AM-positive cells (Fig. 1C). Then, quantitative analysis was performed (Fig. 1D). Statistical result shows a similar trend, indicating that PUN exhibit excellent biocompatibility in both BMMs or RAW 264.7 macrophages and concentrations at or below 80 μmol/L were considered safe, as they did not affect cell viability.

### Evaluation of anti-osteoclast and anti-inflammatory effects of PUN

**Fig. 2:**
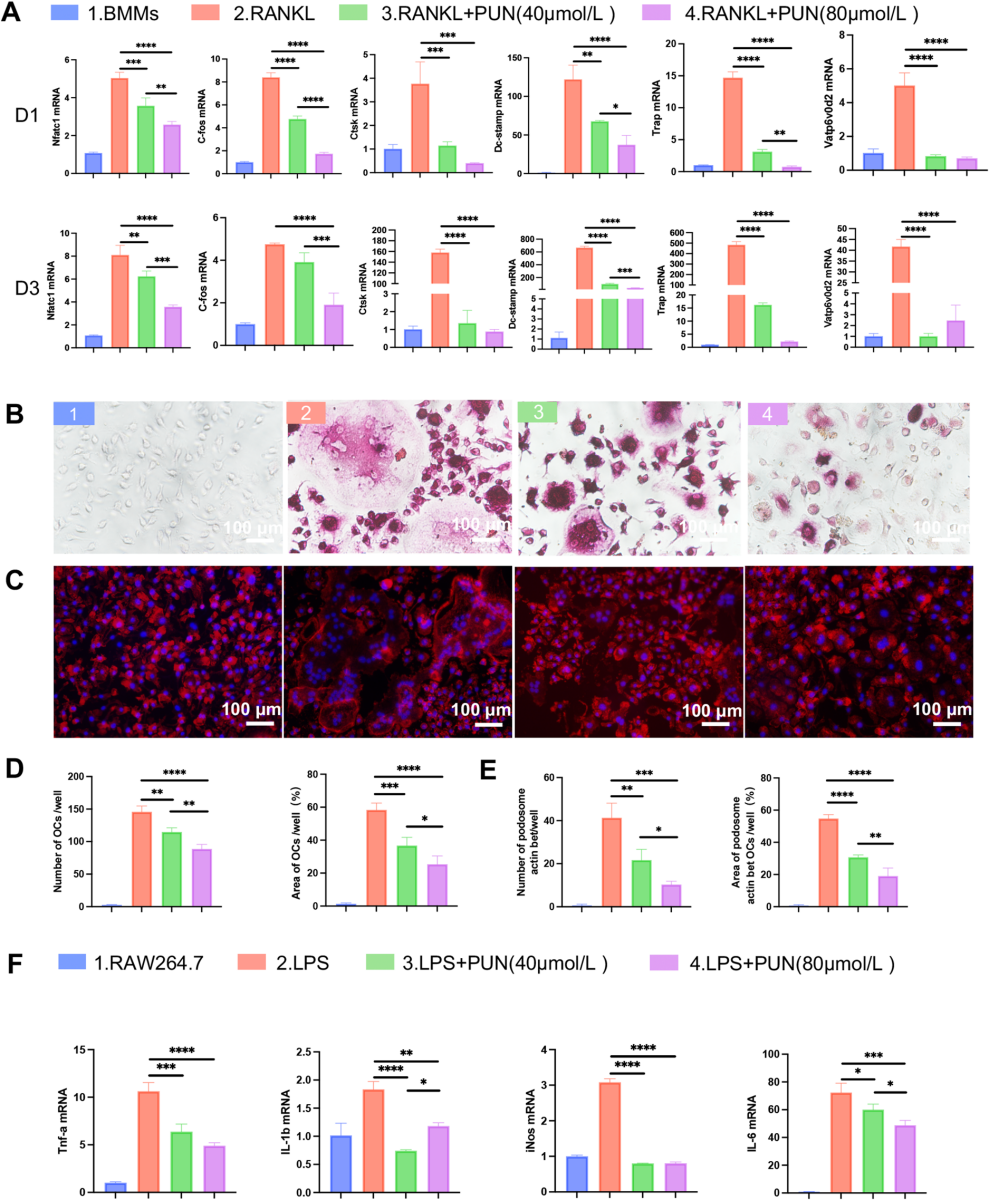
PUN reduced RANKL-induced osteoclastogenesis and LPS-induced inflammation. (**A**) RT-qPCR results of OCs-related genes expression of BMMs, treated with various concentrations of PUN. (**B**) Representative tartrate-resistant acid phosphatase (TRAP) staining images of OCs, treated with various concentrations of PUN. (**C**) TRITC Phalloidin staining images of OCs, treated with various concentrations of PUN. (**D**) Quantitative analysis of the number and area of OCs per well. (**E**) Quantitative analysis of the number and area of podosome actin belts per well. (**F**) RT-qPCR results of anti-inflammatory genes expression of RAW 264.7 macrophages, treated with various concentrations of PUN. (mean ± SD, one-way ANOVA with Tukey’s multiple comparison test, n = 3 independent samples, *p < 0.05; **p < 0.01; ***p < 0.005; ****p < 0.001).

To investigate the effect of PUN on transcript levels in OCs, RT-qPCR was performed to analyze the expression of OC-related genes on Day 1 and Day 3, including Nfatc1, C-fos, Ctsk, Dcstamp, Trap, and Vatp6v0d2. As expected, PUN markedly inhibited the expression of OC-related genes in a dose-dependent manner, including genes associated with OCs fusion, OCs differentiation, and bone resorption function (Fig. 2A). TRAP staining result showed that BMMs could successfully differentiate into mature osteoclasts, containing more than two nuclei, upon treatment with RANKL for 5 days. But this process was disrupted by PUN. After treating BMMs with different concentrations of PUN (0, 40, and 80 μmol/L), the number and area of TRAP-positive OCs were significantly reduced (Fig. 2B and D). We next aimed to determine whether PUN has influence on osteoclast fusion. Immunofluorescence (IF) results showed that PUN inhibited the formation of podosome actin belts in a dose-dependent manner, leading to a decrease of the number and area of podosome actin belts, further indicating that PUN reduced OC precursor cell fusion (Fig. 2C and E). Subsequently, we examined the anti-inflammatory effects of PUN. RT-qPCR results showed that the expressions of Tnf-α, Il-β, iNos and Il-6 were significantly decreased after PUN treatment in RAW 264.7 macrophages (Fig. 2F). In conclusion, these results indicate that PUN strongly inhibits RANKL-induced OC formation, fusion, and function, as well as LPS-induced inflammation responses.

### Transcriptomic analysis of anti-inflammatory and anti-osteoclastic mechanism of PUN

**Fig. 3:**
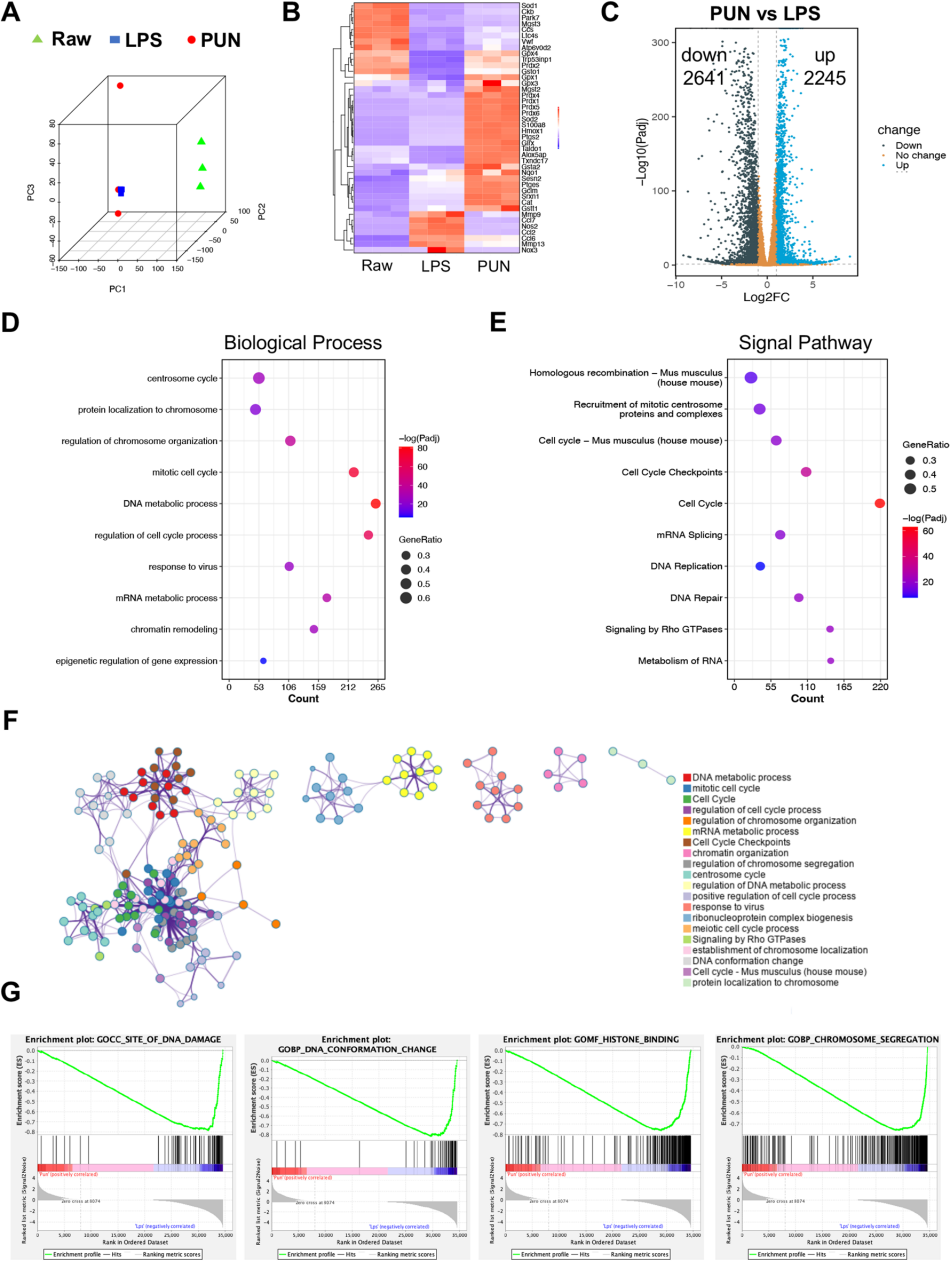
Transcriptomic analysis of anti-inflammatory and anti-osteoclastic mechanism of PUN. (**A**) PCA of the different transcriptomic profiles among the Raw group, LPS group and the PUN-treated group. (**B**) Heatmap Clustered heatmap of expression of pro-inflammatory and antioxidant genes. (**C**) Volcano plots of differentially regulated genes between the LPS group and the PUN-treated group. (**D**) Bubbleplot of biological process enrichment analysis of differentially downregulated genes by PUN. (**E**) Bubbleplot of signaling pathway enrichment analysis of differentially downregulated genes by PUN. (**F**) Biological process network of differentially downregulated genes by PUN. (**G**) GSEA of the expressed genes between the LPS group and the PUN-treated group. (Log2FC ≤ -1 and Padj < 0.05).

To further elucidate the underlying mechanism of PUN’s anti-inflammatory and anti-osteoclastic properties, we performed RNA sequencing. Principal component analysis (PCA) revealed a clear separation between Raw group, LPS group and LPS + PUN group, indicating different transcriptomic profiles (Fig. 3A).The cluster heatmap showed that the expression of various pro-inflammatory genes such as Mmp9, Ccl7, Nos2 was significantly increased after LPS stimulation, while the expression was significantly decreased after PUN treatment (Fig. 3B). The expression of antioxidant genes, including Sod1, Park7, Ccs, and Mgst3 were down-regulated after LPS stimulation, while the expression was significantly increased after PUN treatment. Volcano map showed that 2641 genes were significantly up-regulated and 2245 genes were down-regulated after PUN treatment (Fig. 3C). As shown by RT-qPCR results, transcriptional levels of osteoclast-related genes, including Acp5, Ctsk, Mmp13, Dcstamp, and Atp6v0d2, were also down-regulated, indicating the inhibitory role of PUN. Furthermore, we decided to investigate the potential effects of PUN on biological processes and signaling pathways. We first analyzed down-regulated genes after PUN treatment (log2FC < − 1, Padj < 0.05). Interestingly, we found that Genes related to chromatin spatial structure, were enriched for Gene ontology (GO) terms, including protein localization on chromosomes, regulation of chromosome organization, chromatin remodeling (Fig. 3D). Meanwhile, we observed a high enrichment of signaling pathways involved in acetylation of histones and chromatin remodeling, indicating the underlying regulatory mechanism of PUN to regulate gene expression through chromatin structure. In addition, down-regulated genes after PUN treatment were enriched in signal pathway such as recruitment of mitotic centrosome proteins and complexes, cell cycle, DNA replication, and DNA repair, suggesting that PUN may modulate osteoclastogenesis by affecting cell proliferation (Fig. 3E). Based on selective condition (log2FC < − 2 and Padj < 0.05), 500 genes were identified. Biological process network analysis revealed enrichment in regulation of chromosome organization, protein localization to chromosome, cell cycle, regulation of cell cycle process, meiotic cell cycle process, regulation of chromosome segregation (Fig. 3F), consistent with the above findings.

In addition, gene set enrichment analysis (GSEA) showed that PUN treatment is significantly negatively associated with DNA damage (GO Cellular Component), DNA conformation (GO biological process) and chromosome separation (GO biological process), and histone binding (GO Molecular Function), suggesting a potential role of PUN which may regulate osteoclastogenesis by epigenetic mechanisms(Fig. 3G). Taken together, these data suggest that PUN may exhibit stronger anti-osteoclast and antioxidant effects by influencing the cell cycle, inhibiting metabolic processes, and regulating epigenetically associated pathways.

### Transcriptomic analysis indicated that PUN may regulate chromatin remodeling through BET proteins

**Fig. 4:**
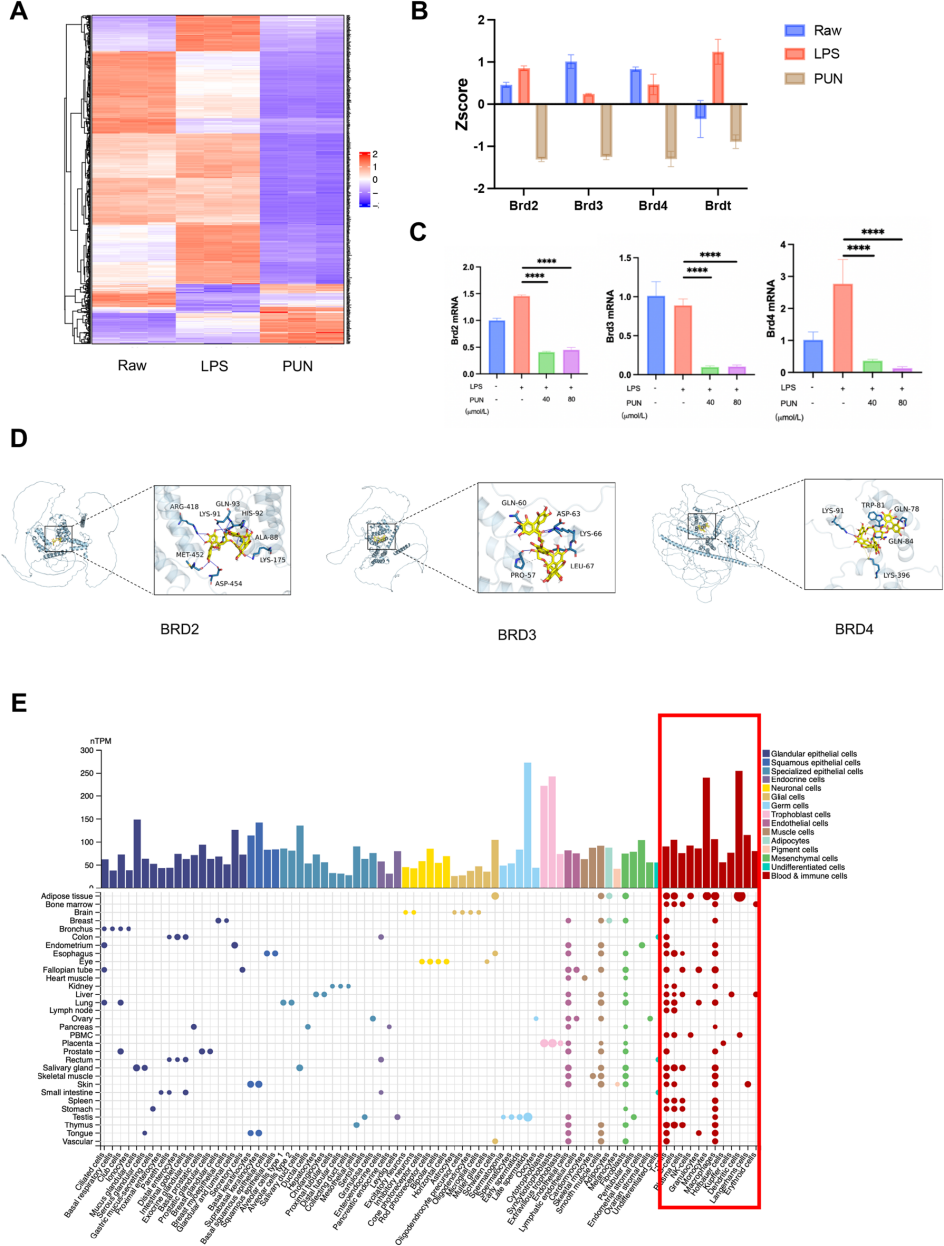
PUN regulates the assembly of histone acetylation-dependent chromatin complexes via BET proteins (**A**) Heatmap Clustered heatmap of expression of anti-inflammatory and antioxidant genes. (**B**) Zscore of BET family genes. (**C**) RT-qPCR results of BET family genes expression of RAW 264.7 macrophages, treated with LPS and various concentrations of PUN. (**D**) Molecular docking suggested stronger binding ability between PUN and BET family proteins. (**E**) TMP (Transcriptional Machineries Profile) of Brd4 in different cell types.

To investigate underlying epigenetic-associated mechanism of PUN, we first generated a heatmap to obtain an overview of transcriptional alteration. As shown in the heatmap, 500 epigenetic related genes changed after PUN treatment (Fig. 4A). Subsequently, we examined whether PUN treatment will affect the expression of BET protein coding genes or not, including Brd2, Brd3 and Brd4. RNA-seq analysis revealed a significant reduction in the expression of these genes after PUN treatment. Consistent with the findings described above, both the enriched biological processes and signaling pathways are highly associated with chromatin remodeling. These observations led us to hypothesize that PUN could suppress the expression of inflammatory genes by regulating BET family members, which play key roles in epigenetic mechanisms. Z-score analysis revealed that expression of BET family genes, including Brd2, Brd3 and Brd4, was significantly suppressed after PUN treatment (Fig. 4B), partially supporting our hypothesis. RT-qPCR analysis showed a similar trend in the expression of BET family members upon treatment with different concentrations of PUN (Fig. 4C).

Based on the molecular docking data, PUN was effectively bound to the active pockets of Brd2, Brd3 and Brd4, with binding energies of − 7.2 kcal/mol, − 7.2 kcal/mol and − 8.2 kcal/mol respectively, indicating that the compound could have strong spontaneous hydrogen bonding and hydrophobic interaction with all of these proteins. Further analysis of three-dimensional interactions reveals the specific binding mode (Fig. 4D). As the database shown, Brd4 is expressed in different tissues, especially in blood and immune cells (Fig. 4E), which prompted us hypothesize that PUN may negatively regulate the expression of downstream inflammatory factors by inhibiting Brd4, thereby mitigating inflammatory osteolysis.

### PUN promoted anti-osteoclast effect via scavenging of ROS and activating endogenous antioxidant systems

**Fig. 5:**
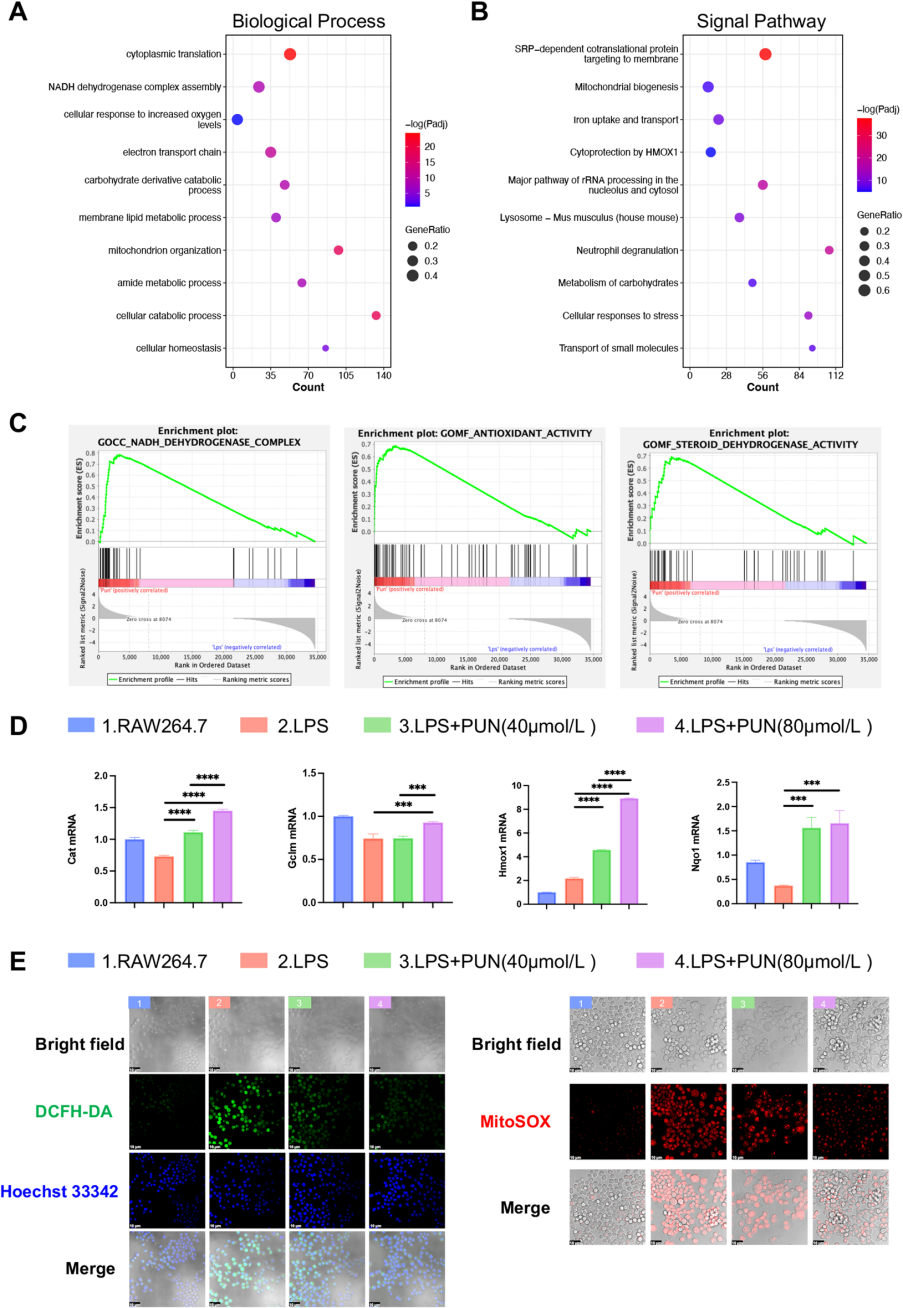

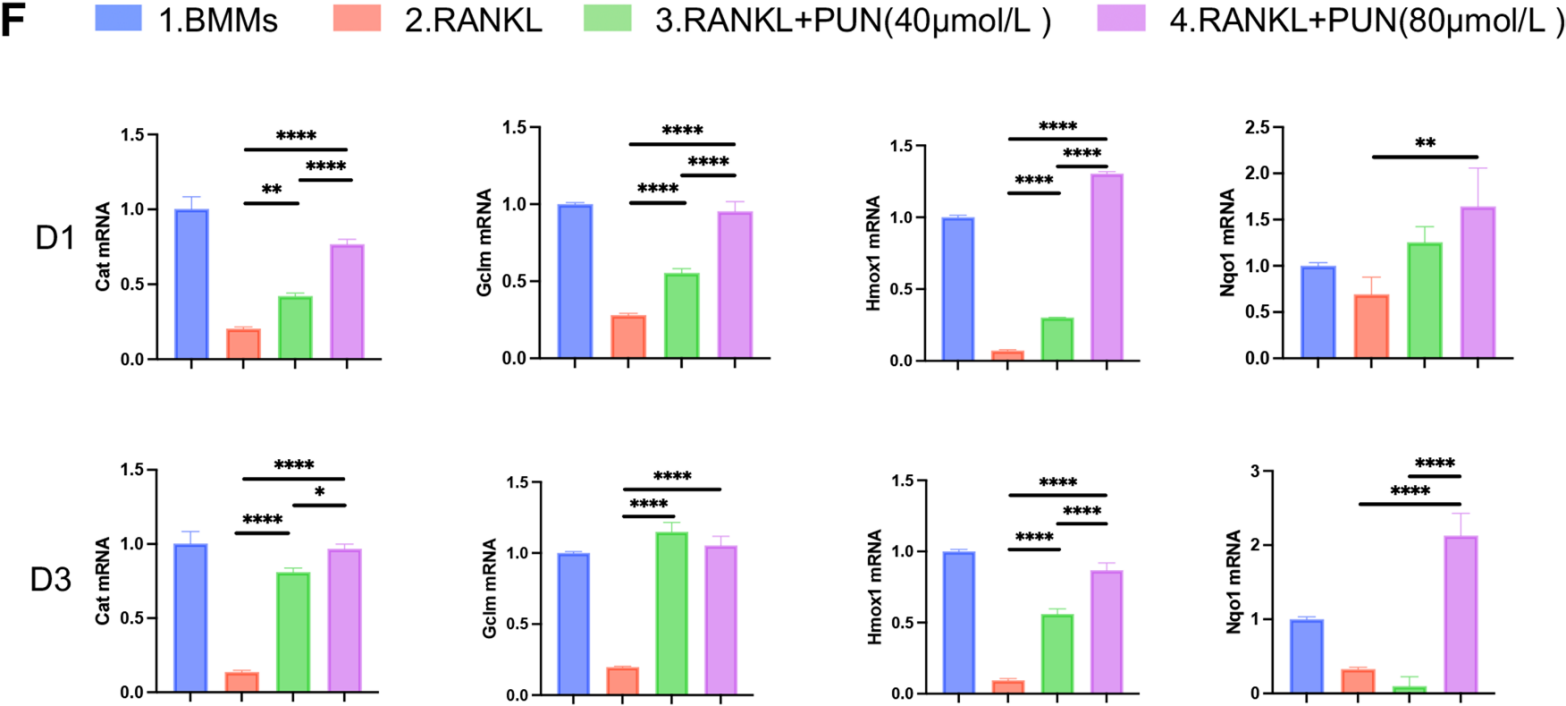
Antioxidant and ROS clearance effects of PUN on osteoclast and LPS-stimulated macrophage. (**A**) Bubbleplot of signaling pathway enrichment analysis of differentially upregulated genes by PUN. (**B**) Biological process network of differentially upregulated genes by PUN. (**C**) GSEA of the expressed genes between the LPS group and the PUN-treated group. (Log2FC ≥ 1 and Padj < 0.05). (**D**) RT-qPCR results of antioxidant genes expression in RAW 264.7 macrophages treated with concentrations of PUN. (**E**) Representative confocal images of RANKL-activated BMMs stained with DCFH-DA and MitoSOX probes. (**F**) RT-qPCR results of antioxidant genes expression in RANKL-activated BMMs treated with concentrations of PUN. (mean ± SD, one-way ANOVA with Tukey’s multiple comparison test, n = 3 independent samples, *p < 0.05; **p < 0.01; ***p < 0.005; ****p < 0.001).

Thus, we decided to discover the potential ROS-associated mechanism underlying PUN treatment. Interestingly, Gene ontology (GO) analysis of upregulated genes showed that significant enrichment of biological processes in PUN-treated group, including Nicotinamide adenine dinucleotide (NADH) dehydrogenase complex assembly, cellular response to increased oxygen levels, electron transport chain, carbohydrate derivative catabolic process, mitochondrion organization and cellular catabolic process (Fig. 5A). Furthermore, signaling pathways related to cytoprotection by heme oxygenase 1 (HMOX1) and metabolism of carbohydrates were also enriched in unregulated genes of PUN- treated group. These findings suggest that PUN may regulate osteoclastogenesis through regulation of cellular respiration and metabolism (Fig. 5B). Consistently, GSEA analysis also support this speculation, revealing significant enrichment of upregulated genes in PUN-treated group for gene sets associated with NADH dehydrogenase complex, antioxidant activity, and steroid dehydrogenase activity (Fig. 5C). To validate the antioxidant effects of PUN, we performed RT-qPCR, which showed that Nrf2 downstream genes (including Hmox1, Nqo1, Cat and Gclm) were up-regulated in a dose-dependent manner after PUN treatment (Fig. 5D). Then, we used DCFH-DA to detect intracellular ROS and MitoSOX for mitochondrial ROS in RAW 264.7 macrophages after treating with different concentrations of PUN (0, 40, and 80 μmol/L) under LPS stimulation for 12 h. Compared with the blank control group, the fluorescence intensity significantly increased after LPS stimulation and the fluorescence intensity was significantly reduced after PUN treatment, and the fluorescence intensity shows a dose-dependent down-regulation manner (Fig. 5E). MitoSOX staining showed a similar trend that mitochondrial ROS was also reduced significantly after PUN treatment and the fluorescence intensity shows a dose-dependent down-regulation manner (Fig. 5E). Similarly, in RANKL-stimulated BMMs treated with PUN, expressions of Hmox1, Nqo1, Cat, and Gclm showed dose-dependent up-regulation on Day 1 and Day 3 (Fig. 5F). This suggests that PUN may exert anti-inflammatory and anti-osteoclastic effects by promoting the activation of endogenous anti-inflammatory and antioxidant products in inflammatory macrophages and osteoclasts.

### Inhibitory effect of PUN on LPS-induced calvarial osteolysis in vivo

**Fig. 6:**
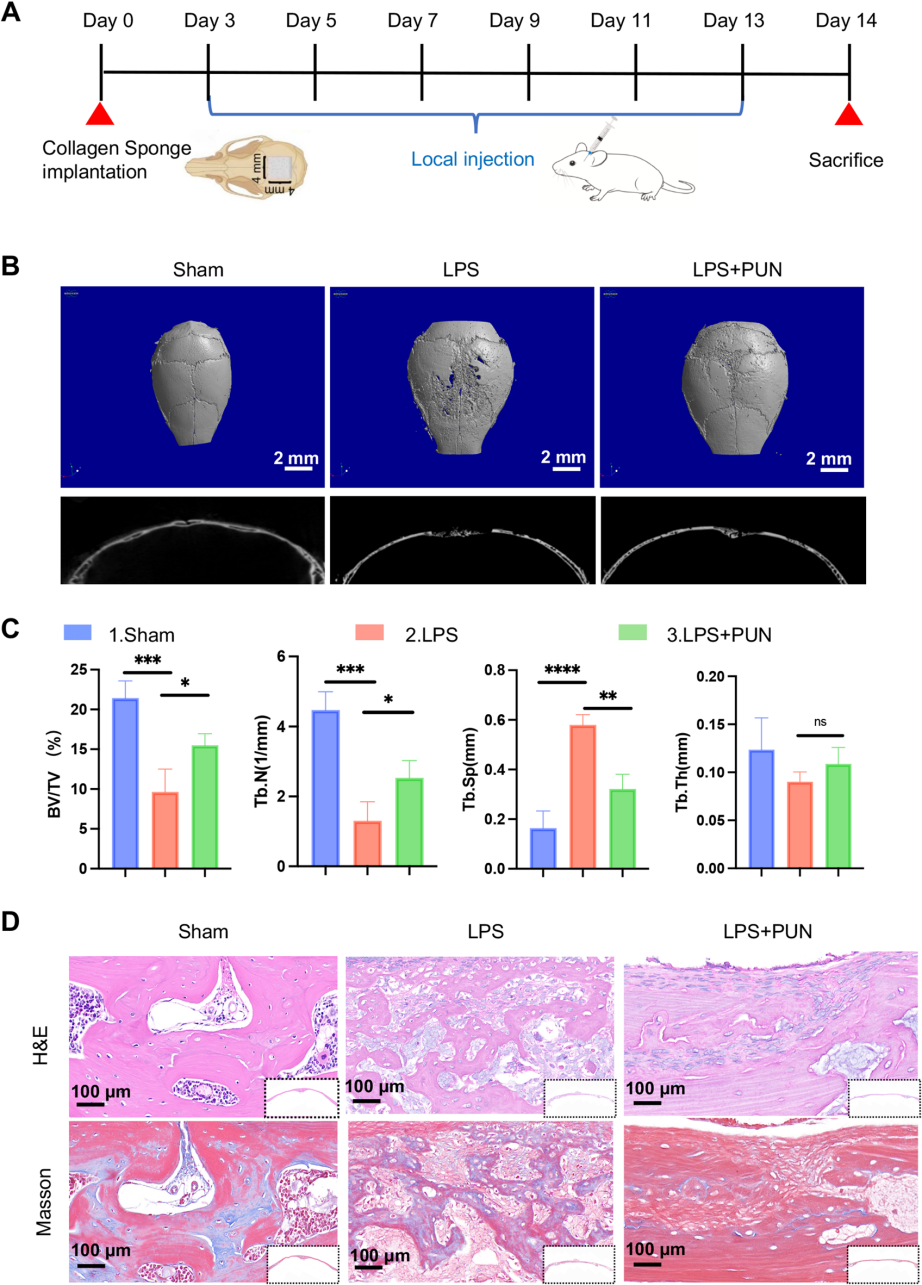
Inhibitory effect of Punicalagin on LPS-induced calvarial osteolysis in vivo. (**A**) Schematic diagram of LPS-induced skull defect in C57BL/6 mice (**B**) Representative images of the micro-CT scanning of skull with three-dimensional reconstruction and two-dimensional view illustrated bone construction after administration with various treatment groups (14 days). (**C**) Quantitative analysis of micro-CT scanning of skull after administration with various treatment groups. (**D**) Representative images of H&E and Masson staining after administration of various treatment groups. (mean ± SD, one-way ANOVA with Tukey’s multiple comparison test, n = 6 independent samples, *p < 0.05; **p < 0.01; ***p < 0.005; ****p < 0.001).

Results described above demonstrated that PUN possesses good biocompatibility and exert a role of anti-osteoclast, anti-inflammatory, ROS scavenging and metabolism-epigenetic effects in vitro. Based on these findings, we next investigated the potential therapeutic effect of PUN in vivo using the murine cranial osteolysis model induced by LPS. The Schematic diagram of the in vivo treatment is presented in Fig. 6A. Using micro-CT 3D reconstruction, we observed that the skull surface in the LPS-treated group exhibited extensive erosion with numerous large and deep absorption pits. In contrast, both the extent and severity of bone damage were significantly reduced in PUN-treated group (Fig. 6B). Data analysis showed that parameters such as bone volume/tissue volume (BV/TV), trabecular number (Tb.N) and trabecular separation (Tb.Sp) were significantly improved in PUN group compared with LPS group (Fig. 6C). We then performed histological staining to confirm the protective effect of PUN on bone absorption and inflammation in vivo. Consistent with the micro-CT results, H&E staining and Masson staining showed extensive osteolysis caused by local injection of LPS, while PUN reduced the extent of osteolysis (Fig. 6D). Taken together, these results suggest that PUN can attenuate LPS-induced osteolysis by inhibiting bone resorptive and inflammatory activity and activating endogenous anti-inflammatory systems.

## Discussion

The occurrence of inflammatory osteolysis has multiple causes. However, regardless of the etiology or cellular background, abnormal osteoclast differentiation is a key factor in its pathogenesis^7,41^. As specialized terminally differentiated cells, osteoclasts serve as the principal effectors of bone resorption and play crucial roles in bone development, growth, repair, and remodeling. During inflammatory osteolysis, microenvironmental alterations significantly modify the activity and differentiation status of chronic inflammatory macrophages, leading to excessive production of inflammatory cytokines that subsequently promote macrophage-to-osteoclast differentiation^42,43^. The impact of inflammatory cytokines on osteoclastogenesis and function has been instrumental in developing therapeutic strategies for focal osteolysis, yielding agents that either directly inhibit osteoclasts or indirectly suppress their activity through cytokine blockade. Nevertheless, a substantial proportion of inflammatory arthritis patients exhibit resistance to current therapies. This underscores the need for deeper mechanistic insights into inflammasome-mediated signaling pathways, encompassing both cytokine-dependent and -independent mechanisms, to optimize treatment strategies for inflammatory osteolysis. In this study, we employed in vitro culture of osteoclast precursor cells and a murine model of inflammatory osteolysis to clarify the therapeutic effect and potential mechanism of PUN in inflammatory osteolysis.

First, we systematically evaluated the biocompatibility and safe concentration range of PUN using CCK-8 assays and Calcein-AM/PI double staining at varying concentrations and treatment durations. Our results confirmed that PUN exhibited no significant cytotoxicity at 80 μmol/L, establishing a safe concentration threshold for subsequent in vitro and in vivo experiments. TRAP staining revealed that PUN suppressed OC formation in a concentration-dependent manner. Further investigations demonstrated that PUN not only impaired OC differentiation but also disrupted cytoskeletal organization—a critical structural determinant of osteoclastic bone resorption. Notably, actin dynamics, particularly the arrangement and remodeling of F-actin, are essential for OC activity and resorptive capacity^44^. Our findings align with this premise, confirming that PUN effectively inhibits OC function. RT-qPCR analysis indicated that PUN significantly downregulated key osteoclastogenic genes, which are involved in OC fusion, differentiation, and bone resorption. These results suggest that PUN exerts its inhibitory effects at the transcriptional level, modulating pathways critical for osteoclastogenesis. We further assessed the anti-inflammatory properties of PUN in LPS-induced macrophages. RT-qPCR analysis demonstrated marked reductions in the expression of pro-inflammatory factors following PUN treatment. Collectively, these findings indicate that PUN effectively attenuates both RANKL-induced osteoclastogenesis and LPS-induced inflammatory responses.

Compared with LPS group, the expression of Cat, Hmox1, Gclm, Nqo1 and other anti-inflammatory and antioxidant genes was up-regulated after PUN treatment. Then, we use high-throughput sequencing to explore the potential mechanism of the anti-inflammatory effect of PUN. The volcano map revealed that PUN significantly regulated the expression of thousands of genes, including down-regulated osteoclast genes and up-regulated antioxidant genes, which not only verified its antioxidant properties, but also suggested that antioxidant may be one of the important ways to inhibit osteoclast cell formation. The results of GO enrichment analysis showed that biological processes such as protein localization on chromosomes, chromosome organization regulation, chromatin remodeling, mitotic cell cycle, cell cycle process regulation, and DNA metabolism were significantly correlated with PUN’s therapeutic mechanism. Meanwhile, pathway enrichment analysis also revealed that the signaling pathways closely related to the antioxidant and anti-osteoclast effects of PUN were highly enriched, involving histone acetylation, DNA damage, chromatin remodeling and chromatin separation, suggesting that PUN may play a role by influencing the cell cycle, inhibiting metabolic processes and regulating epigenetic pathways. In addition, GSEA also found significant enrichment of NADH dehydrogenase complex, antioxidant activity and steroid dehydrogenase activity related genes, indicating that the antioxidant capacity was significantly regulated after PUN treatment. Therefore, we then conducted further tests using ROS probes, and the results showed that both intracellular ROS and mitochondrial ROS were significantly reduced.

Interestingly, we found that PUN may exert its regulatory effects through epigenetic mechanism for several epigenetic pathways enriched markedly. Therefore, we hypothesize that PUN may regulate expression of inflammatory-associated genes through epigenetic alteration. By overlapping the differentially expressed genes with the list of human epigenetic regulators, we identified significant changes in BET family members. Both Z-score analysis and RT-qPCR data showed that the expression of BET family members, including Brd2, Brd3 and Brd4, were significantly suppressed following PUN treatment. Molecular docking suggested stronger binding ability between PUN and Brd4. As a key epigenetic regulator, Brd4, which is highly expressed in blood and immune cells, could selectively recognize and bind histones with acetylated marks, thereby regulate transcription via RNA polymerase II (Pol II) ^45^. Disruption of Brd4 is associated with development of multiple bone-related disease ^46^. In this context, our study suggest that PUN may downregulate inflammatory-associated -associated genes by inhibiting Brd4 in an epigenetically mediated manner, thereby inhibiting the process of inflammatory osteolysis. Combined with RNA-seq analysis, we speculate that PUN may exert its effects by modulating the spatial structure of chromatin. Nevertheless, additional experiments will be requied to validate this hypothesis.

In conclusion, we demonstrate the potential therapeutic effect of PUN in the treatment of inflammatory osteolysis. The inhibitory effect of PUN can be attributed to the following mechanisms:

1. Suppression of osteoclastogenesis: PUN suppresses differentiation, formation, and fusion of OCs.
2. Anti-inflammatory activity: PUN attenuates inflammatory responses by promoting expression of anti-inflammatory factors.
3. Antioxidant effects: PUN enhance the expression of antioxidant genes in inflammatory condition, thereby mitigating oxidative stress.
4. Epigenetic regulation: PUN may modulate osteoclastogenesis in an epigenetic-associated manner, particularly via regulating the transcriptional activity of Brd4, thus regulating spatial structure of chromatin to regulate expression of downstream genes, including inflammatory genes.

Despite these encouraging preclinical observations, several limitations need to be acknowledged in future research and clinical translation. First, the low oral bioavailability of PUN (approximately 12%)^47^ poses a critical barrier to its in vivo efficacy, as sufficient concentrations may not reach bone tissues through conventional administration routes. Second, current evidence^48,49^ is primarily derived from in vitro cell models and limited animal experiments; the therapeutic effect and safety profile of PUN in large animal models or human clinical trials have not been validated. Finally, the synergistic effects of PUN with other bioactive components or conventional therapies (e.g., bisphosphonates) for inflammatory osteolysis have not been explored, and the long-term effects on bone metabolism and systemic homeostasis require further investigation.

**Fig. 7:**
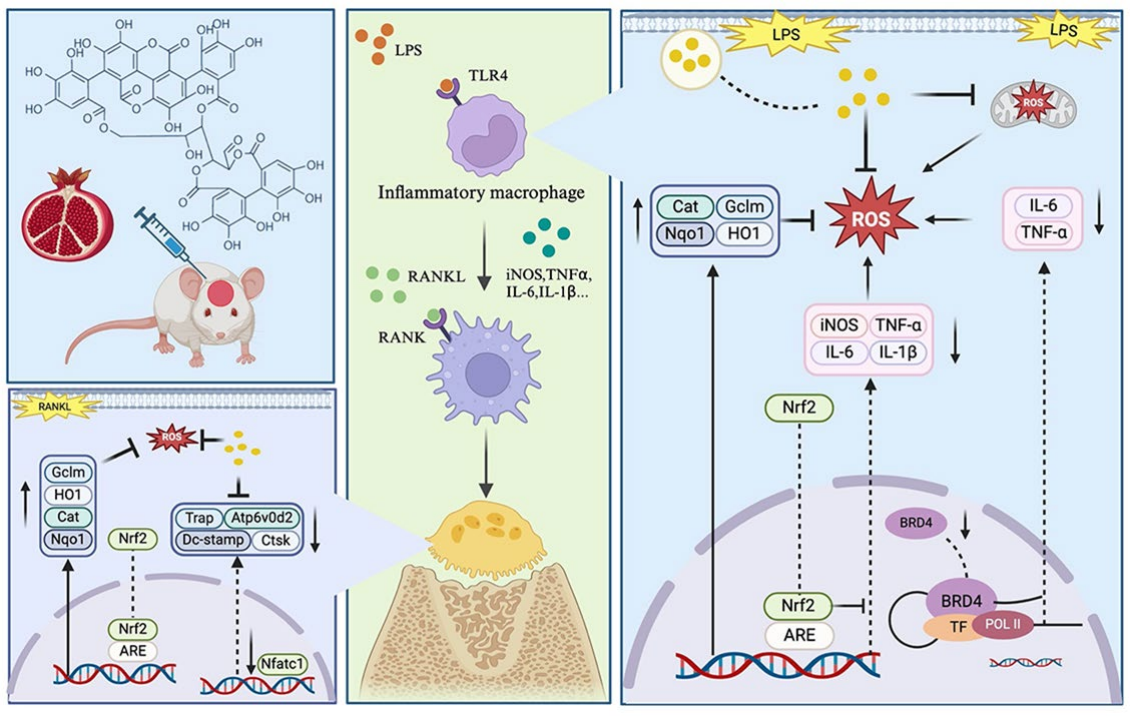
Schematic representation of the potential application of Punicalagin to treat inflammatory osteolysis.

Taken together, the potent anti-inflammatory and antioxidant effects, as well as epigenetic regulatory effects of PUN, endow it with strong potential for the treatment of inflammatory osteolysis (Fig. 7). Limitations of the present study include the lack of in vivo validation of the Brd4-dependent epigenetic regulatory axis, etc. The further research focus on employing conditional Brd4 knockout mouse models and biophysical binding assays, as well as exploring the pharmacokinetic profile of PUN in vivo to optimize its therapeutic potential.

## Materials and methods

### Reagents and antibodies

Recombinant mouse RANKL and macrophage colony-stimulating factor (M-CSF) were purchased from R&D (Minneapolis, MN, USA). Penicillin and streptomycin were purchased from from Gibco BRL (Gai- thersburg, MD, USA). Minimal essential medium alpha (α-MEM) was purchased from YuanPei (Shanghai, China). Escherichia coli-derived lipopolysaccharide (LPS) was obtained from InvivoGen (San Diego, CA, USA). Fetal bovine serum (FBS) was purchased from Avantor (Radnor, PA, USA). The Cell Counting Kit-8 (CCK-8) was purchased from Dojindo Molecular Technology (Rockville, MD, USA). Calcein/PI and 2-(4-Amidinophenyl)-6-indolecarbamidine dihydrochloride (DAPI) were obtained from Beyotime Institute of Biotechnology (Shanghai, China). The Prime Script RT reagent Kit and TB Green Premix Ex Taq II were obtained from Takara Biomedical Technology (Beijing, China). TRAP was obtained from JoyTech (Zhejiang, China). Primary antibodies against Hmox1, Nqo1, and Keap1 were purchased from Cell Signaling Technology (CST, Danvers, MA, USA). Punicalagin was purchased from Selleck (Houston, Texas, USA).

### Cell culture

Bone marrow-derived macrophages (BMMs) were freshly isolated from the long bones of six-week-old male C57/BL6 mice. Then, the isolated cells were cultured in a complete medium of α-MEM (supplemented with 10% heat-inactivated FBS, 100 U/mL penicillin/streptomycin, and 30 ng/mL MCSF). RAW 264.7 macrophages were grown in DMEM (supplemented with 10% heat-inactivated FBS, 100 U/mL penicillin/streptomycin). The cell cultures were maintained in a humidified atmosphere with 5% CO2 at 37 °C.

### Cell counting kit-8 assay

To assess cell viability after treatment with different concentrations of PUN. BMMs or RAW 264.7 macrophages were seeded in a 96-well plate and at a density of 1 × 10^5^ cells/well, then stimulated with various concentrations of PUN for 24, 48, and 72 h. The Cell Counting Kit-8 (CCK-8) (ApexBio, USA) assay was performed at 24, 48, and 72 h according to the manufacturer’s protocol.

### Quantitative real-time PCR analysis

To analyze the mRNA level of antioxidant-related and anti-inflammatory genes, RAW 264.7 macrophages were cultured in 6-well plates at a density of 5 × 10^5^ cells/well and divided to four groups (Group 1: Blank; Group 2: LPS only; Group 3: LPS 100 ng/mL + PUN 40 μmol/L; Group 4: LPS 100 ng/mL + PUN 80 μmol/L), then stimulated with LPS (100 ng/mL) plus various concentrations of PUN (0, 40 and 80 μmol/L) for 24 h. To analyze the expression of antioxidant-related and osteoclast-related genes, BMMs were seeded in 6-well plates at a density of 2 × 10^5^ cells/well and divided to four groups (Group 1: Blank; Group 2: RANKL only; Group 3: RANKL 50 ng/mL + PUN 40 μmol/L; Group 4: RANKL 50 ng/mL + PUN 80 μmol/L), then cultured with MCSF (30 ng/mL), RANKL (50 ng/ mL) and various concentrations of PUN (0, 40 and 80 μmol/L) for another 1 and 3 days. The total RNA of each group was extracted by using an Axygen RNA Miniprep Kit (Axygen, Union City, CA, USA), according to the manufacturer’s instructions. TB Green Premix Ex Taq II was used for a real-time PCR (RT-qPCR) assay on an ABI 7500 Sequencing Detection System (Applied Biosystems, Foster City, CA). 5 μL of TB Green, 3 μL of ddH2O, 1 μL of cDNA, 0.4 μL of each primer and 0.2 μL ROX Dye2 were mixed to establish a 10 μL reaction system. Cycling conditions were set as 40 cycles (95 °C for 5 s and 60 °C for 30 s). The melting curves were checked to verify the amplification specificity. Data were normalized to GAPDH expression using the 2^−ΔΔCT^ method. Error bars represent the standard deviation (S.D.) of technical triplicates. The sequences of the primers are listed in Table 1.

**Table 1:** Sequences of primers for real-time quantitative polymerase chain reaction.

Gene	Forward primer sequence (5′–3′)	Reverse primer sequence (5′–3′)
*C-fos*	CCAGTCAAGAGCATCAGCAA	AAGTAGTGCAGCCCGGAGTA
*Ctsk*	GGGAGAAAAACCTGAAGC	ATTCTGGGGACTCAGAGC
*DC-stamp*	AAAACCCTTGGGCTGTTCTT	AATCATGGACGACTCCTTGG
*Trap*	CTGGAGTGCACGATGCCAGCGACA	TCCGTGCTCGGCGATGGACCAGA
*Vatp6v0d2*	AAGCCTTTGTTTGACGCTGT	TTCGATGCCTCTGTGAGATG
*Tnf-α*	GCCTCTTCTCATTCCTGCTTGTGG	GTGGTTTGTGAGTGTGAGGGTCT
*IL-1β*	TCGCAGCAGCACATCAACAAGAG	AGGTCCACGGGAAAGACACAGG
*iNos*	ACTCAGCCAAGCCCTCACCTAC	TCCAATCTCTGCCTATCCGTCTCG
*Brd2*	AATGGCTTCTGTACCAGCTTTAC	CTGGCTTTTTGGGATTGGACA
*Brd3*	GGGCGAAAGACTAACCAACTG	GAAAGGCCAGGCAAACTGATG
*Brd4*	GTGAGAAGCTAGGCCGTGTAG	AGGCAGGACCTGTTTCAGAGT
*Cat*	GGAGGCGGGAACCCAATAG	GTGTGCCATCTCGTCAGTGAA
*Gclm*	AGGAGCTTCGGGACTGTATCC	GGAAACTCCCTGACTAAATCGG
*Hmox1*	AGGTACACATCCAAGCCGAGA	CATCACCAGCTTAAAGCCTTCT
*Nqo1*	AGGATGGGAGGTACTCGAATC	TGCTAGAGATGACTCGGAAGG
*Gapdh*	GGTGAAGGTCGGTGTGAACG	CTCGCTCCTGGAAGATGGTG

### Tartrate-resistant acid phosphatase (TRAP) staining assay

BMMs were seeded in 96-well plates at a density of 8000 cells/well and cultured with MCSF (30 ng/mL), RANKL (50 ng/mL), and different concentrations of PUN (0, 40, and 80 μmol/L). The formation of OCs maybe visible at fourth or fifth day. The cells were fixed with 4% paraformaldehyde (PFA) for 30 min and washed three times with PBS. Mix the TRAP ingredients (1–0002–5 mL; JoyTech, Zhejiang, China) as recommended by the manufacturer and add 100 μl of TRAP solution to each well. Then the plates were incubated in a 37 °C oven for 30 min to 1 h. Optical microscope was used for image capture of TRAP-positive cells with three or more nuclei. The number and area of OCs were quantitatively analyzed using ImageJ software (NIH, Bethesda, MD, USA).

### Detection of podosome actin belt

BMMs culture is the same as TRAP staining. After the maturation OC was formed, the cells were fixed and permeabilized, then covered with TRITC Phalloidin (1:100; CA1610; Solarbio, Beijing, China) and incubated at room temperature for 30 min away from light. After that, the nuclei were stained with DAPI for 5 min. The cells were washed with PBS three times, then observed via CLSM. The number of podosome actin belts was quantified by ImageJ software (NIH, Bethesda, MD, USA).

### RNA sequencing

The potential mechanisms of antioxidant and anti-inflammatory effect of PUN were explored by RNA sequencing. RAW 264.7 macrophages were seeded in 6-well plate, the control group received no special treatment, while the experimental groups were respectively stimulated with LPS (100 ng/mL) and/or PUN (80 μmol/L) for 24 h. Total RNA was collected using TRIZOL reagent. The gene expression levels were detected by Biomarker Technologies (Beijing, China). Differentially expressed genes have a *p* adjust value < 0.05 as well as a log2 fold change <  − 1. Gene ontology (GO) enrichment analysis and PPI network analysis were performed using Metascape (https://www.metascape.org). GSEA was performed following the developer’s protocol (https://www.broad institute.org/gsea/). The normalized enrichment score, nominal *p* value, FDR and q value are presented to determine the significance of enrichment level. The RNA-seq data are from three biological samples.

### Molecular docking

In order to better confirm the interaction between PUN and BET proteins including Brd2, Brd3, and Brd4, molecular docking was performed. Chemical structure of PUN was retrieved from the PubChem database (https://pubchem.ncbi.nlm.nih.gov/), and the obtained SDF files were converted to PDB format using Open Babel 2.3.2 software. The receptor protein was acquired from the UniProt database, followed by modification via hydrogenation and charge neutralization using AutoDockTools software. Both the processed receptor proteins and PUN were converted to PDBQT format individually. Molecular docking between the receptor proteins and PUN were performed using AutoDock Vina 1.1.2, and the docking results were analyzed with the Protein–Ligand Interaction Profiler (PLIP) tool.

### Detection of reactive oxygen species (ROS)

We used fluorescein dichlorodihydrodiacetate (DCFH-DA) probes (S0033S; Beyotime Biotech- nology, China) to detect intracellular ROS and MitoSOX (40778ES50; Yeasen, China) for mitochondrial ROS detection. RAW 264.7 macrophages were inoculated in confocal culture dishes and divided to four groups (Group 1: Blank; Group 2: LPS only; Group 3: LPS 100 ng/mL + PUN 40 μmol/L; Group 4: LPS 100 ng/mL + PUN 80 μmol/L). After that, the cells were incubated with serum-free culture solution containing 10 μM DCFH-DA for 20 min or 5 μM MitoSOX for 10 min at 37℃. After three times washing with PBS, images were captured by CLSM.

### LPS-induced calvarial osteolysis model

We established the murine calvarial osteolysis model to evaluate the in vivo therapeutic effect of PUN on inflammatory response and bone resorption, based on the previous reports^50–53^. The Animal Care and Experiment Committee of Ninth People’s Hospital Affiliated to Shanghai Jiao Tong University School of Medicine (SH9H-2025-A1529-1) approved the animal protocol. We conform that all methods were performed in accordance with the relevant guidelines and regulations of Animal Research: Reporting of In Vivo Experiments (ARRIVE) Guidelines. All animals were purchased from Shanghai Xipuer-Bikai Laboratory Animal Co., Ltd. In brief, 8-week-old C57/BL6 male mice (approximate weight 25 ± 2 g) were randomly divided into three groups (n = 6 per group): (1) Sham group (with PBS injection); (2) LPS group (10 mg/kg body weight of LPS and PBS injection); (3) PUN group (10 mg/kg body weight of LPS and 50 mg/kg body weight of PUN injection). Mice were anesthetized with isoflurane (induction: 2.5–3%, maintenance: 1–1.5%), then a PBS or LPS-soaked collagen sponge (4 mm × 4 mm × 2 mm) was implanted into the median of the skull of mice to induce inflammatory bone loss. Three days after the collagen sponge implantation, LPS or LPS + PUN was injected subcutaneously at the calvarial apex every other day for a total of six injections. 11 days later, the mice were humanely euthanized using CO₂ asphyxiation (flow rate: 20% chamber volume per minute) and subsequent cervical dislocation to ensure death, following the guidelines approved by the Institutional Animal Care and Use Committee (IACUC). The entire skull was removed, washed with PBS and then immobilized in 4% PFA for 48 h for imaging and histological analysis.

### Micro-computed tomography

The calvaria harvested from the different groups were analyzed using a high-resolution micro-CT scanner (micro-CT, Sky- scan1176, USA Bruker) with source voltage of 70 kv, source current of 200 uA, image pixel size of 8.9658 um, and a fixed exposure time of 350 ms. The microstructure indicators of bone volume/tissue volume (BV/TV), trabecular number (Tb.N), trabecular separation (Tb.Sp), and trabecular number (Tb.Th) were analyzed by the program CTAn (Bruker microCT, Kontich, Belgium). The three-dimensional (3D) and two-dimensional (2D) images were reconstructed with ctvox (Bruker microCT, Kontich, Belgium).

### Histological and immunohistochemical analysis

After micro-CT scanning, the samples were decalcified in 10% EDTA (pH = 7.4) for 4 weeks, then embedded in paraffin. Histological sections were prepared for hematoxylin and eosin (H&E) and Masson staining. The images of stained slices were captured under a high-quality microscope (Leica DM4000B). The number of OCs and TRAP-positive multinucleated OCs per field (Oc.S/BS) were calculated.

### Statistical analysis

Prism 9.0 statistical software package (GraphPad Software Inc, San Diego, CA, USA) was used to analyze the results and the data were presented in the form of mean ± standard deviation (SD) uniformly. After the homogeneity test, the two-tailed unpaired Student’s t-test was used to analyze the two groups. One-way analysis of variance (ANOVA) with Tukey’s post hoc tests were used for multiple group comparisons. Significant differences were determined to be at *p < 0.05 and **p < 0.01.

## Supplementary Information

Below is the link to the electronic supplementary material.

Supplementary Material 1

## Data Availability

The data that support the findings of this study are available from the corresponding author upon reasonable request. The datasets generated and/or analyzed during the current study are available in https://www.ncbi.nlm.nih.gov/sra/PRJNA1328197, BioProject ID: PRJNA1328197.
