# Supplementary material for: Punicalagin with anti-inflammatory activities affects Brd-4 mediated chromatin remodeling for attenuating inflammatory osteolysis
Source: Sci Rep. 2026 Mar 10;16:12948. doi: 10.1038/s41598-026-41262-3 (PMC13096185; doi:10.1038/s41598-026-41262-3)
Supplement: Supplementary file 1 — Supplementary Material 1 [file 41598_2026_41262_MOESM1_ESM.pdf]

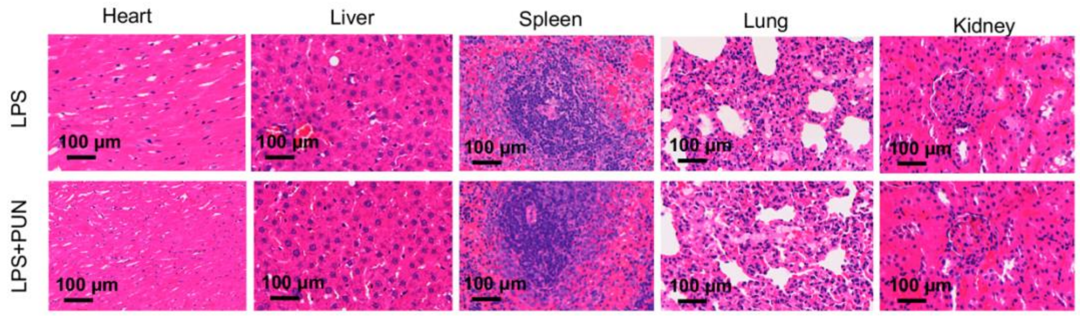

Fig. S1 Representative images of H&E staining of heart, liver, spleen, lung and kidney of LPS and LPS+PUN group.
